# Challenges faced by patients with dyslipidemia and systemic arterial hypertension in Brazil: a design of the patient journey

**DOI:** 10.1186/s12872-022-02669-8

**Published:** 2022-05-21

**Authors:** Jose Rocha Faria-Neto, Carlos Yarleque, Luiz Fernando Vieira, Eliane Naomi Sakane, Raul D. Santos

**Affiliations:** 1 School of Medicine, Pontificial Catholic University of Parana (PUCPR), Curitiba, Brazil; 2 Research, Development and Medical, Upjohn - A Division of Pfizer, Lima, Peru; 3 Research, Development and Medical, Pfizer Upjohn, Sao Paulo, Brazil; 4 Heart Institute (InCor) University of Sao Paulo Medical School Hospital, São Paulo, Brazil; 5 Academic Research Organization, Hospital Israelita Albert Einstein, São Paulo, SP Brazil

**Keywords:** Systemic arterial hypertension, Dyslipidemia, Patient journey, Prevalence, Diagnosis, Treatment

## Abstract

**Background and objective:**

Non-communicable diseases like systemic arterial hypertension (SAH) and dyslipidemia are poorly studied in terms of patient journey aspects. This semi-systematic review provides evidence synthesis for the management of SAH and dyslipidemia in Brazil and also discusses challenges faced by patients at the local level along with a suggested care approach by local experts.

**Methods:**

A semi-systematic review using both structured literature databases (Embase and Medline) and unstructured scientific records (WHO, IPD, MOH and Google) on hypertension and dyslipidemia in the English language from 2010 to 2019 was performed by reviewers. After two-level screening based on pre-defined criteria, patient journey touchpoints and prevalence information were extracted from the included articles. Data gaps were bridged through the insights of local experts.

**Results:**

Prevalence of hypertension and dyslipidemia in Brazil were 23% and 40.8%, respectively. Awareness of dyslipidemia was found in a larger proportion (58.1%) than in SAH (22.2%). Similarly, screening for hypertension (97%) and dyslipidemia (55.4%) were found to be effective, while treatment was (62.9%) and (30.0%) for hypertension and dyslipidemia, respectively.

**Conclusion:**

There were important gaps on patient awareness and treatment of dyslipidemia and hypertension. Limited patient education, regional disease distribution, and treatment allocation, along with limited resources for diagnosis and treatment are the key challenges.

**Supplementary Information:**

The online version contains supplementary material available at 10.1186/s12872-022-02669-8.

## Introduction

Cardiovascular diseases (CVDs) are the major cause of death worldwide with an estimated global mortality of 17.9 million in 2016 [1], which is expected to increase to 23.6 million in 2030 [2]. Systemic arterial hypertension (SAH) and dyslipidemia are the primary risk factors for cardiovascular complications [3]. SAH is a multi-system complex disease with an estimated prevalence of 37.3% and 22.9% in developing and industrialized countries, respectively [4], and accounts for 45% of cardiac deaths and 51% of stroke-related deaths [5]. Dyslipidemia is characterized by the presence of elevated blood low-density lipoprotein cholesterol (LDL-C) or high triglycerides with or without low levels of high-density lipoprotein cholesterol (HDL-C) [6]. High LDL-C levels are an independent risk factor for coronary artery disease [7]; elevated LDL-C levels of ≥ 100 mg/dL increased the risk of CVD-related death by 30%, whereas LDL-C levels ≥ 130 mg/dL resulted in > 50% increased risk in coronary heart disease-related death [8]. Raised cholesterol causes nearly 33% of global ischemic heart diseases accounting for nearly 2.6 million deaths every year [9].

Studies in Brazil have shown a nearly 30% prevalence of hypertension in the adult population, corresponding to nearly 36 million patients [10], and the prevalence of dyslipidemia in some regions in Brazil is nearly 32.7% [11]. Data on prevalence show that diagnosis of dyslipidemia is more frequent in women (15.1%) compared with men (9.7%) [12]. A similar pattern was observed through Vigitel (a phone health survey conducted by the Brazilian Ministry of Health), where the prevalence of dyslipidemia in women and men was 25.9% and 18.8%, respectively [13]. Regarding age, hypertension is more common in individuals of age 50–59 years than in those who are 20–29 years old [3], and a higher prevalence of elevated cholesterol has been reported in the Brazilian population aged 45 years or older and with low education [11].

The coexistence of dyslipidemia and hypertension has been reported in 50 to 80% of patients and their synergistic effects result in an increased risk of CVDs, which is greater than the sum of individual risks [14, 15]. Although the causal association is yet to be proven, epidemiological studies have shown that low HDL-C levels are associated with a twofold increase in fatal CVD risk [16].

Despite the major contribution of SAH and dyslipidemia in cardiovascular morbidity and mortality, data regarding their nationwide prevalence, awareness, screening, diagnosis, treatment, adherence, and control are scarce. Most of the studies in the Brazilian population are region-specific, thus limiting their applicability to the population of the entire country. For non-communicable chronic diseases (NCDs), an understanding of the patient journey from a patient’s perspective can help improve healthcare systems to address the unmet needs of patients as well as increase patient participation and disease management [17]. Given the sparse information on screening, diagnosis, and treatment of SAH and dyslipidemia in Brazil, the present review aims to provide a situational analysis of the current management of these conditions and to identify challenges in care delivery at various levels to help strategize the optimization of the patient journey as a whole.

## Methods

### Search strategy and selection criteria

A structured search using Medline, Embase followed by unstructured search using resources like World Health Organization (WHO), Ministry of Health (MOH) websites, Incidence and Prevalence Database (IPD) and Google was performed from Dec 10, 2019 by reviewer 1. The search terms included words related to patient journey touchpoints, clinical condition (SAH and Dyslipidemia) as well as context (Brazil). The detailed search strategy is given in Appendix. Original research, systematic review, meta-analyses articles in English from 2010 to 2019 were retrieved (except for one study that was presented in 2019 but published in full detail in 2022). After duplicate removal, first-level screening was done by reviewer 1 based on relevance. Non-relevant records were excluded during level-2 screening by reviewer 2 based on exclusion criteria, that included age < 18 years, case reports, letter to editors, articles with no patient journey data (prevalence, awareness, screening, diagnosis, treatment, adherence, and control), written in a non-English language, articles with non-representative study sample or study from other than Brazil region [18]. Also, this review excluded studies with specific groups of patients (elderly, pregnant women, co-morbidities). Any disagreements about the inclusion of any record were resolved through discussion between both reviewers. An additional file shows detailed search strategy (see Additional file 1).

Hypertension was defined as systolic blood pressure (BP)  ≥ 140 mm Hg and/or diastolic BP ≥ 90 mm Hg [19]. Dyslipidemia was defined as total cholesterol of  ≥ 5 mmol/L or ≥ 200 mg/dL [20]. Disease control was defined as the proportion of patients achieving a target BP of ≤ 140/90 mm Hg or cholesterol of ≤ 5 mmol/L or ≤ 200 mg/dL with treatment.

### Data extraction and synthesis

Data extraction included study year, sample size, prevalence, awareness, screening, diagnosis, treatment, adherence, and control data points (in percentages) and associated challenges from both patient’s and health system’s angle. Data gaps were addressed by supplementing with local studies and anecdotal data from local experts. Quantitative data for prevalence, awareness, screening, diagnosis, treatment, adherence, and control were synthesized through weighted mean or simple mean.

### Statistical analysis

Quantitative data on the patient journey stages for hypertension and dyslipidemia were extracted and analyzed from the included studies. Weighted means were calculated in the case of multiple data points for a patient journey stage.

### Availability of data and materials

Study data is available if requested to the corresponding author (RDS) by e mail.

## Results

### Study selection

**Fig. 1:**
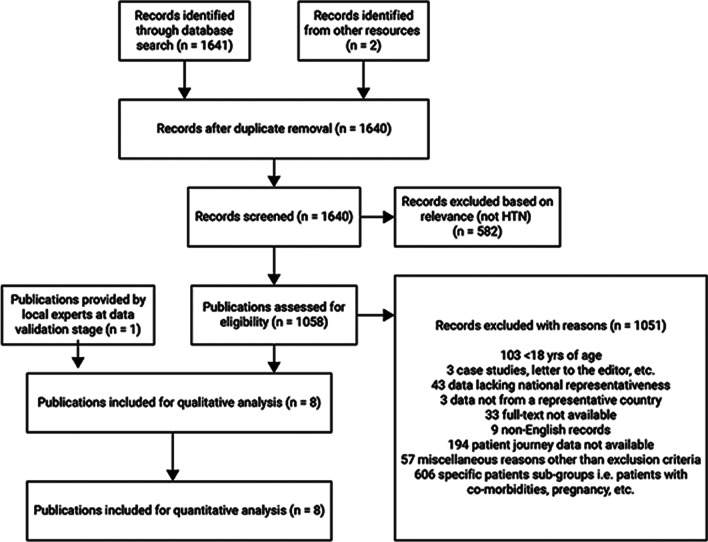
PRISMA flow diagram for systemic arterial hypertension in Brazil

**Fig. 2:**
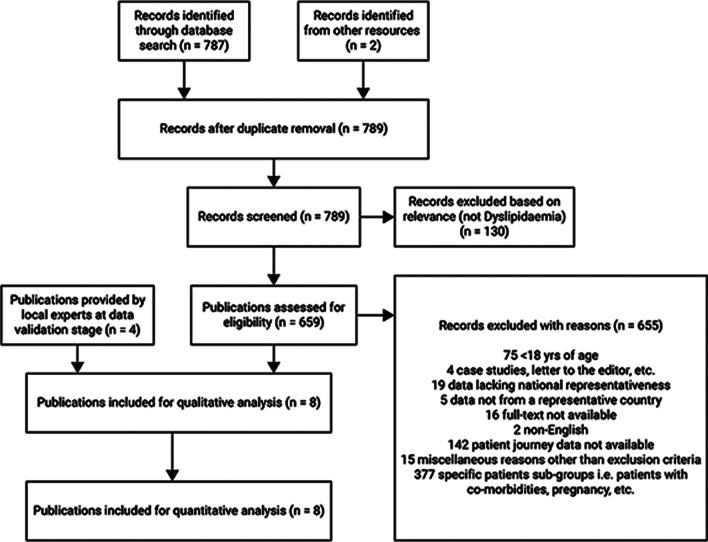
PRISMA flow diagram for dyslipidemia in Brazil

Overall, 2428 articles were retrieved from the structured search and 4 were obtained from the unstructured search. After the abstract screening, 103 articles were identified as relevant. Six articles were obtained from external experts. These were manually screened for inclusion and exclusion criteria, and finally, 16 full-text articles and one anecdotal evidence were included. The characteristics of the included articles/records and PRISMA flow diagrams for SAH and dyslipidemia are given in Table 1, Figs. 1 and 2, respectively.

**Table 1:** Characteristics of included studies

S. No.	Author	Year	Sample size	Prevalence (%)	Awareness (%)	Screening (%)	Diagnosis (%)	Treatment (%)	Adherence (%)	Control (%)
*Systemic arterial hypertension*										
1	Lamelas P [21]	2019	5553	52.5	64.7			62.7		23.4
2	Global Status Report on Noncommunicable Diseases 2014 [22]	2014	20,27,64,000	23						
3	Malta [11]	2018	60,202	32.3	21.4					
4	Picon [23]	2012	122,018	28.7	20.6					
5	Malachias [24]	2016		32.5				11.4–77.5		10.1–35.5
6	Lotufo [25]	2015				97		81.4		
7	Barbosa [26]	2019	7260	47						60
8	Vigitel Brazil 2018 [27]	2018	52,395				24.7			
9	Regional Experts								50	
*Dyslipidemia*										
1	Malta [11]	2019	8534	32.7						
2	Borgo [28]	2019	318	42.3						
3	Goncalves [29]	2019	60,202		11.03		12.5			
4	Vigitel Brazil 2016 [13]	2016	53,210		22.6					
5	Lotufo [12]	2017	60,202			55.4	12.5			
6	do Nascimento [30]	2018	8803					24.6	93.5	
7	Bernardi [31]	2019/2022	1451							29.8
8	Lotufo [32]	2016	15,105	45.5	58.1^#^			42.3		58.3

^#^This value was the only one considered for the final (summary) analysis for “awareness” data point for dyslipidemia

### Summary of findings

**Fig. 3:**
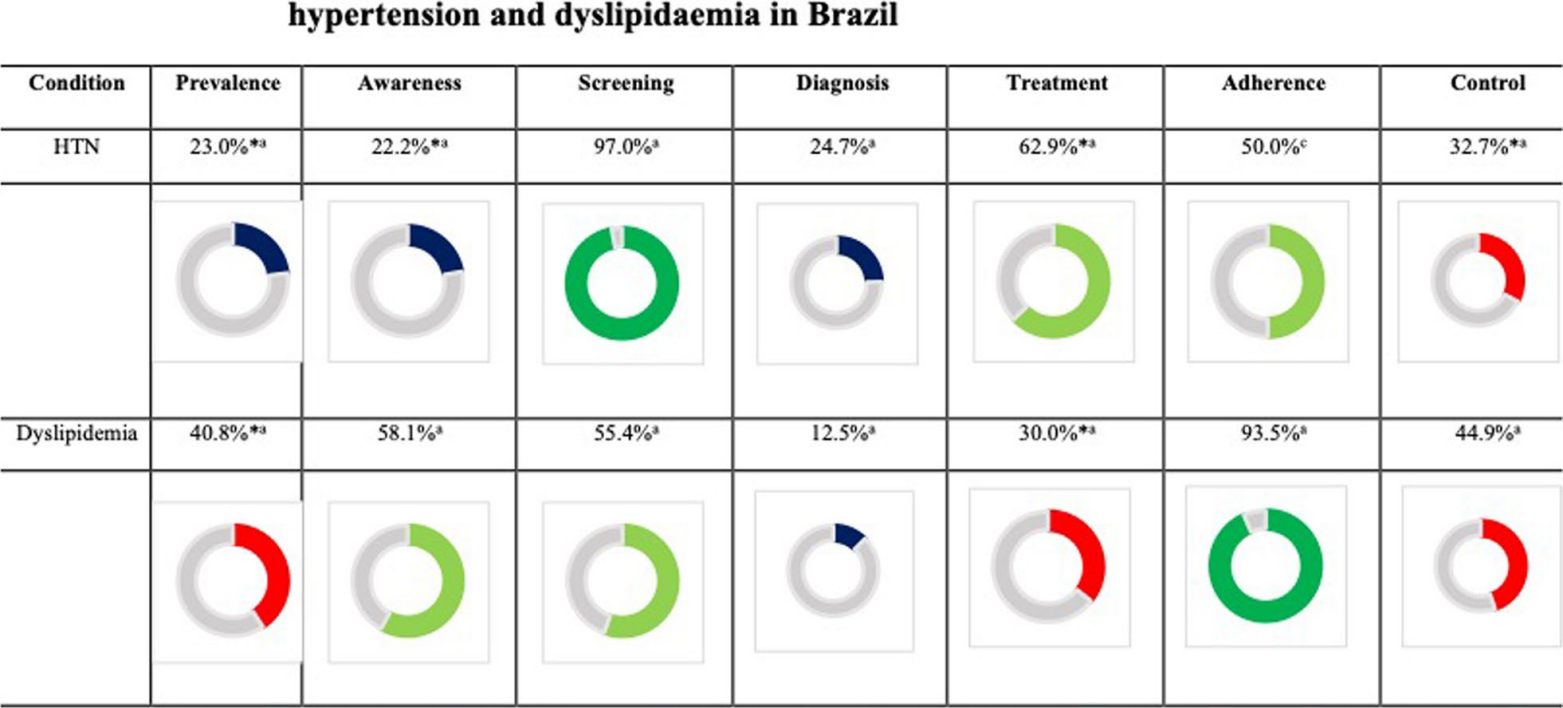
Summary of findings from the included studies on hypertension and dyslipidemia in Brazil. *Weighted average; ^a^Peer-reviewed publication; ^b^Scientific literature + expert opinion; ^c^Expert opinion only. Here navy blue, red, light green, and dark green colored doughnuts are indicative of percentage ranges ≤ 25%, 25–50%, 50–75%, and ≥ 75%, respectively. Health literacy: 68%^a^

The prevalence of hypertension in Brazil was 23% [11, 21–24, 26], and a low patient awareness (22.2%) was observed on SAH [11, 21, 23]. Strategies for screening hypertension were found to be effective, and 97% of the population was screened [25]; however, the patient diagnosis was suboptimal (24.7%) [27]. Most of the patients diagnosed with hypertension followed pharmacological treatment (62.9%) [21, 24, 25]; however, only half of them (50%) adhered to the treatment, as opined by local experts. As a result, the target outcomes were achieved by only one-third of the patients (32.7%) [21, 24, 26]. Dyslipidemia was observed in 40.8% of the population [11, 28, 32] and awareness regarding the disease was found in a larger proportion (58.1%) than in SAH [32]. Although more than half of the patient population was screened (55.4%) [12], only a small number of patients received proper diagnosis (12.5%) [12, 29], thus suggesting the need for expanding the reach of diagnostic techniques. The percentage of patients receiving treatment was low (30.0%) [30, 32], which may reflect the challenges in providing equitable access to dyslipidemia treatment for patients across the country, or the lack of prescription by the physicians who overlook the importance of lipids control. Maybe as a result, the proportion of patients who achieve the cholesterol levels recommended by the Brazilian Society of Cardiology [32] is low (29.8%) [33]. A previous study described overall cholesterol control rates around 58%, but they observed that the control of LDL-C was markedly lower in those patients with higher risk (9.4%) [29]. Only one of the retrieved studies provided adherence data regarding statin use and reported it to be above 90%. The authors felt like this high rate could have been overestimated because it was a self-reported survey and the question regarded only the week before the appointment [31]. Overall, patients with hypertension had a better level of screening, diagnosis, and treatment than patients with dyslipidemia. However, awareness and adherence were found to be higher in patients with dyslipidemia compared to those in patients with hypertension. The graphical visualization of the summary of findings can be seen in Fig. 3.

## Discussion

The Brazilian healthcare system is segmented into public and private subsystems. The public subsystem named the Sistema Único de Saúde (SUS) provides free and universal healthcare access to all citizens, covering nearly 75% of the population. Most of the patients get treated by general practitioners at first and are unable to get cardiology consultations unless there are any established coronary diseases, core comorbidities or secondary complications. On the other hand, in the private subsystem, the patient can go directly to the specialist they want to see irrespective of their health condition. Approximately 30% of the Brazilian CVD patients directly visit private care specialists because of better access to diagnostic tests, quicker appointments, and better customized care. The approach to treatment in public and private institutions is also different. For example, risk factors control is limited in the case of primary care, general practitioners, or family physicians. Some studies worldwide suggest that private specialist clinics have been more successful in delivering care and achieving the recommended goals when treating the patients, and local publications are in line with these observations [33–37].

### Challenges with patient journey touchpoints

Combined results of several studies and awareness campaigns conducted by the Brazilian Society of Cardiology showed that nearly one-third of the Brazilian population is hypertensive and general awareness is low [38]. Low patient awareness could be attributable to low patient education and prevailing social inequalities, which affect access to and use of health services and medications, as well as adherence to medical prescription [39]. In Brazil, nearly half of all CVD-related deaths in patients aged < 65 years are attributable to poverty, social inequalities, and low educational status [40–42]. Furthermore, awareness regarding the benefit of a healthy lifestyle is limited, and we observe the adoption of unhealthy diets, low physical activity, and high consumption of alcohol and tobacco especially in low-income communities [40]. It is important that both patients and health care professionals belonging to the public health system [43, 44] receive continued education and updates from the latest available clinical practice guidelines from the Brazilian Society of Cardiology, and that they understand the importance of adopting the guidelines and achieving the goals in daily practice.

A limited sense of urgency in the Brazilian population for timely diagnosis and management of CVDs due to the asymptomatic nature of the conditions was observed. As a result, although the number of screened patients with SAH and dyslipidemia was high, it did not convert into effective care. Moreover, the diagnosis of complex diseases that require molecular diagnosis, like familial hypercholesterolemia, is hindered by the limited availability of specialized laboratories [45, 46].

The percentage of patients receiving treatment for SAH was higher than that for dyslipidemia. This could be attributable to better screening and diagnosis of SAH and limited access to lipid lowering medications in certain regions in Brazil. Limited access to healthcare and regional variations in treatment protocols further complicates the scenario.

Addressing adherence-related issues is fundamental for reducing CVD-related morbidity and mortality. In Brazil, pharmacological treatment guidelines developed by international or local societies/associations for CVD prevention are not strictly followed. Reasons for low patient adherence in SUS primary healthcare were limited access, forgetfulness, and adverse effects of medicines, with several studies reporting unintentional non-adherence in most patients [30].

### Challenges identified in government initiatives

There are programs run by the Brazilian government ensuring free-of-cost access to selected medications [47]. This initiative works rather well owing to the involvement of drugstore chains; however, there are limitations regarding the type of medications available as part of such programs. Furthermore, there are no specific programs in either public or private health systems to monitor and optimize patient adherence. A few public healthcare professionals in several regions of Brazil visit patients [48] at home to follow up on chronic and genetic diseases, but data about the effectiveness of this initiative are limited.

### Proposed healthcare model

The proposed healthcare model for CVD care in Brazil focuses on 3 pillars: patient education, guideline adoption, and overall optimization of the patient journey.

Population-based approaches in awareness campaigns should be prioritized over individual-based approaches for the prevention, detection and control of CVDs, for it is known that early detection may translate into better control. Blood pressure(BP) and cholesterol levels measurements could be made mandatory at issuance places for government documents, like passports, driver’s license, marriage certificate, or other security cards, contributing to a substantial increase in screening and awareness [21]. The healthcare providers should explain the importance of routine check-ups and the negative consequences of non-adherence and delayed treatments of SAH and dyslipidemia to the patients. This could help develop a sense of urgency and proactivity, leading to better compliance and adherence to treatment. Also, encouraging the population to adopt healthy lifestyle habits, including healthier diets and exercise programs, along with the guidance about the medications, can help lower the risk of the patients.

Secondly, strategic adoption of up-to-date clinical practice guidelines with customization based on the regional context could enhance healthcare professionals’ compliance. Healthcare programs should be designed to align with the needs of the most affected populations, such as the older patients, which may not be the target of current initiatives. Not only medications guidelines should be followed. For example, reinforcing dietary portfolios, such as reduction in sodium intake and the dietary approaches to stop hypertension (DASH) type of diet [49], should be considered as an early measure with the potential to decrease the progression from prehypertension to hypertension.

**Fig. 4:**
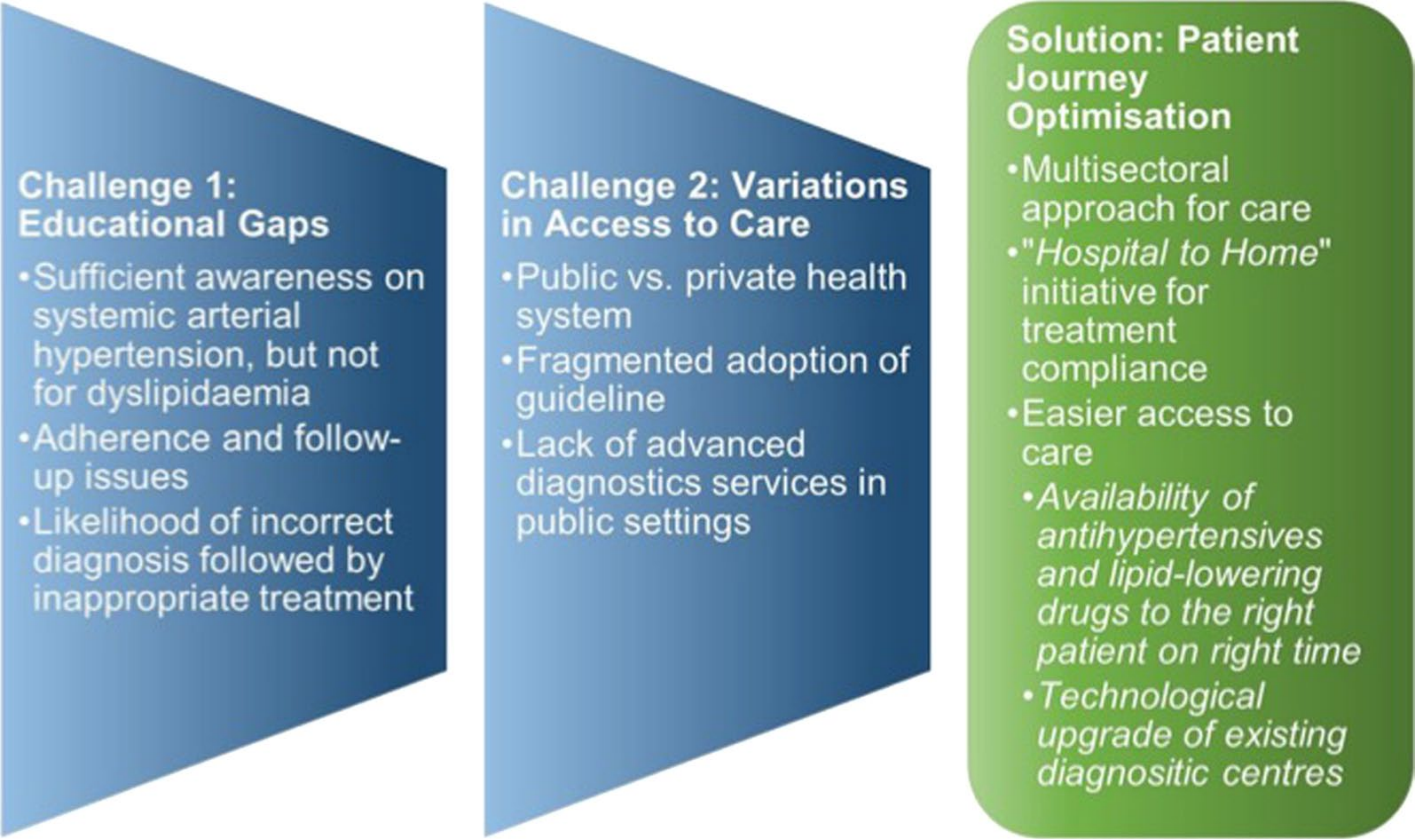
Key patient journey challenges and proposed solutions for patients with cardiovascular diseases in Brazil

The third and most important pillar of our proposed healthcare model is the overall optimization of the patient journey in SAH and dyslipidemia. Non-physician healthcare workers should be trained to screen for the diseases, helping overcome the shortage of physicians in primary care centers. ‘Hospital to home’ initiative should be followed aiming to improve treatment compliance, resulting in better patient quality of life, and outcomes [50]. This initiative focuses on smooth transitioning from hospitals to homes by providing psychological and medication-related care to patients based on their requirements and has been effective in reducing rehospitalization by as much as 61% in the high-risk adult population [51]. Besides ensuring adequate treatment from the healthcare system, it is important to provide easy access and availability of antihypertensive and lipid-lowering medications for patients with SAH and dyslipidaemia, demanding an active role of pharmacy professionals. Also, technological upgrades of existing medical centers can help improve the management of these diseases. For example, the World Heart Federation recommends point-of-care testing for measuring cholesterol in less developed regions [52]. Hand-held echocardiography in primary care centers should be encouraged over conventional echocardiography as a prioritization tool for patients with heart disease because of its resource-friendliness. As a step forward, integrating echocardiographic tracking and distance interpretation through telemedicine can help resolve the problems of late diagnosis and long queues for patients requiring specialized care [53]. Partnering with academia could help in building up the healthcare workforce capacity and skills at individual, family, and community levels. Clinical outcome analysis should be performed with a patient-centered care delivery focus, with the possible incorporation of new technologies to help facilitate this delivery. Engaging patients in health care decision-making could contribute to integrating personalized care concepts. Lastly, the government could devise innovative financing models comprising funds to healthcare facilities for conducting local surveys regarding patient journey mapping, and also for establishing a universal and sustainable healthcare system. Figure 4 summarizes the challenges and proposed solutions related to CVD patient journey in Brazil.

The prevalence of hypertension found in Brazil (23%) was similar to that Latin America (20.2%), while higher in Unites States (30.5%). For hypertension prevalence in Santiago (Chile), Buenos Aires (Argentina), and Barquisimeto (Venezuela), ranged from 24 to 29% slighter higher, whereas in Quito (Ecuador), Bogota ´ (Colombia), Mexico City (Mexico), and Lima (Peru) varied from 9 to 13% was lower compared to Brazil [54]. Approximately (63.0%) of adults with hypertension were aware of their disease condition in Latin America while awareness (22.2%) is low in Brazil. Treatment and control rates of hypertensive patients were lower in Brazil (48.7%) and (43.3%) as compared to Latin America (62.9%) and (60%), respectively [55].

## Limitations

This semi-systematic review has a few limitations. A major limitation of this study is due to publication bias; the exclusion criteria of only full-text publications, non-clinical study types, such as case reports and letters to the editor, studies conducted on specific patient subgroups will not be captured in the analysis. Inclusion of only English language studies results in a language bias.

## Conclusion

The scope of this semi-systematic review was to design the patient journey to devise ways to improve patient awareness and diagnosis for SAH and dyslipidemia in Brazil. The major challenges to SAH and dyslipidemia management in Brazil included patient education, regional disease distribution and medicament allocation, as well as limited resources for proper diagnosis and treatment. To overcome these, we propose strict adoption of the latest treatment guidelines and intensification of patient awareness campaigns about healthy diet and lifestyle modification, the importance of screening for early disease diagnosis, and the benefits of treatment adherence. Also, optimization of the patient journey could involve the implementation of a multisectoral care delivery model focused on medication adherence, availability of skilled resources to meet local care demands, technological integration, and patient-centric policy initiatives. To combat the disease various strategies should be designed to improve the patient journey at each touchpoint of their journey in the healthcare system such as: awareness, screening, diagnosis, treatment, and adherence. These data call for prominent actions to implement effective intervention programs with aim to improve SAH and dyslipidemia.

## Supplementary Information

**Additional file 1.** Detailed Search Strategy (key terms used for search with Boolean operators and inclusion-exclusion criteria).

## Data Availability

The datasets analyzed are available in the bibliographic section. No patient data/file was used in the current study. In addition, data from local experts supporting findings of this study will be available on request from the corresponding author.

## Abbreviations

SAH: Systemic arterial hypertension
CVDs: Cardiovascular diseases
LDL-C: Low-density lipoprotein C
HDL-C: High-density lipoprotein C
NCDs: Non-communicable diseases
WHO: World Health Organization
MOH: Ministry of Health
IPD: Incidence and prevalence database
BP: Blood pressure
SUS: Sistema Único de Saúde
DASH: Dietary approaches to stop hypertension
