# Supplementary material for: Challenges faced by patients with dyslipidemia and systemic arterial hypertension in Brazil: a design of the patient journey
Source: BMC Cardiovasc Disord. 2022 May 21;22:237. doi: 10.1186/s12872-022-02669-8 (PMC9124411; doi:10.1186/s12872-022-02669-8)
Supplement: Supplementary file 1 — Additional file 1. Detailed Search Strategy (key terms used for search with Boolean operators and inclusion-exclusion criteria). [file 12872_2022_2669_MOESM1_ESM.docx]

# Additional file 1: Detailed Search Strategy (key terms used for search with Boolean operators and inclusion-exclusion criteria)

| S. No. | Search String |
| --- | --- |
| 1. | (hypertension OR blood pressure OR hypertensives) AND (epidemiology OR prevalence OR incidence OR national OR survey OR registry) AND (awareness OR knowledge OR health literacy OR screening OR diagnosis OR diagnosed OR undiagnosed OR treatment OR treated OR untreated OR control OR controlled OR uncontrolled OR adherence OR compliance OR adhere OR therapy OR non-adherence) AND Brazil |
| 2. | (dyslipidaemia OR hypercholesterolaemia OR cholesterol OR triglycerides OR LDL) AND (epidemiology OR prevalence OR incidence OR national OR survey OR registry OR statistics) AND (health literacy OR screening OR awareness OR knowledge OR treated OR treatment OR diagnosis OR undiagnosed OR diagnosed OR therapy OR controlled OR control OR uncontrolled OR adherence OR adhere OR compliance) AND (Mexico OR Brazil OR Argentina OR Latin America) |
